# Cytogenetic and Molecular Analyses of Philadelphia Chromosome Variants in CML (chronic myeloid leukemia) Patients from Sindh using Karyotyping and RT-PCR

**DOI:** 10.12669/pjms.314.7261

**Published:** 2015

**Authors:** Ikram Din Ujjan, Anwar Ali Akhund, Muhammad Saboor, Muhammad Asif Qureshi, Saeed Khan

**Affiliations:** 1 Dr. Ikram Din Ujjan, PhD. Department of Pathology, Liaquat University of Medical and Health Sciences, Hyderabad, Pakistan; 2 Prof. Dr. Anwar Ali Akhund, PhD. Department of Pathology, Isra University Hyderabad, Sindh - Pakistan; 3 Dr. Muhammad Saboor, PhD. Baqai Institute of Hematology, Baqai Medical University, Karachi, Pakistan; 4 Dr. Muhammad Asif Qureshi, MBBS (Dow), PhD (Glasgow-UK). Assistant Professor, Department of Pathology, Dow University of Health Sciences, Karachi, Pakistan; 5 Dr. Saeed Khan, BSc, MSc, PhD, Post-doc (USA). Assistant Professor, HOD Molecular Pathology, Dow University of Health Sciences, Karachi, Pakistan

**Keywords:** Philadelphia chromosome, BCR-ABL, Chronic Myeloid Leukemia, cytogenetic analyses, Sindhi population

## Abstract

**Objective:**

To determine the frequency of Philadelphia chromosome (Ph) and its variants in chronic myeloid leukemia (CML) cases at a tertiary care hospital of Sindh.

**Methods:**

The study was conducted at the Department of Pathology, Liaquat University of Medical and Health Sciences, Jamshoro and Isra University Hospital, Hyderabad during May-to-September 2014. Bone marrow and peripheral blood samples from a total of 145 diagnosed cases of CML were collected. Cytogenetic analyses were performed using karyotyping as per the International System for Human Cytogenetic Nomenclature guidelines. All karyotypic images were analyzed using the Cytovision software. In order to identify BCR-ABL transcripts, RT-PCR was performed. Statistical analysis of the data was done using SPSS-version-21.0.

**Results:**

Of the 145 samples, a total of 133 (91.7%) were positive for the Ph (Ph+) while 12 (8.3%) were negative for the Ph (Ph-). Of the 133 Ph+ samples, standard karyotypes were noted in 121 (91%), simple variants in 9 (6.7%) and complex variants in 3 (2.3%) of the samples. All the Ph+ samples (n=133) showed BCR-ABL positivity. Of the 12 Ph- samples, a total of 7 (58.3%) were BCR-ABL-positive and 5 (41.6%) were BCR-ABL-negative.

**Conclusion:**

Frequency of the Ph was found to be of 90.9% in CML patients using a highly sensitive technique, the RT-PCR. Cytogenetic abnormalities were at a lower frequency. Cytogenetic and molecular studies must be conducted for better management of CML cases. These findings could be very useful in guiding the appropriate therapeutic options for CML patients.

## INTRODUCTION

Chronic myeloid leukemia (CML) is a myeloproliferative disorder characterized by the presence of the Philadelphia chromosome (Ph) which is the derivative chromosome 22 of the translocation t(9; 22) (q34.1; q11.2) (OMIM 608232). Due to this rearrangement, the break-point cluster region (*BCR*) gene at position 22q11^1^,^2^ is juxtaposed to the c-Abelson (*ABL1*) gene at 9q34, resulting in the *BCR-ABL1* fusion gene (OMIM 151410), which encodes a constitutively active tyrosine kinase protein. The identification of this abnormality is important for the diagnosis of the diseases determined by the WHO Tumor Classification and for treatment purposes. The first therapeutic choice, tyrosine kinase inhibitors, has shown great therapeutic efficacy.^2^ The Ph is detected by G-band karyotyping in around 90% of CML patients among whom 5–10% may have variant chromosome types.^3^-^5^

Variant Ph chromosomes are characterized by the involvement of another chromosome in addition to chromosome 9 or 22. It can be a simple type of variant when only one additional chromosome is involved, or complex, in which two or more chromosomes, besides chromosomes 9 and 22, take part in the translocation.^6^,^7^ Variant Ph breakpoints occur in hotspots across the genome, usually in the G-light bands, within the cytosine and guanine richest parts of the genome.^8^ However, the mechanism of variant Ph generation and the molecular bases of biological differences between classic Ph and variant Ph chromosomes are not fully understood.^9^ Recently, Albano *et al*.^10^ reported a study they performed on gene expression profiling using microarrays to identify some of these differences.^10^

The prognostic significance of variant Ph chromosomes has already been discussed^11^ and it has been shown that the variant aberration does not impact on cytogenetic or molecular responses or even on clinical outcome. Five variant Ph chromosomes are distinguished from additional chromosomal abnormalities or clonal evolution that derives disease progression. The clonal evolution is a reflection of a genetic instability that characterizes the transition to advanced phase.^12^ In this situation, i(17q), a second Ph and +8 are frequently found.^1^

A study of local literature showed only a few studies have been conducted and reported, hence there is an urgent need to conduct more studies to explore the cytogenetic and molecular abnormalities in CML cases in our local population of Sindh.

## METHODS

### Sample collection

This cross-sectional study was conducted at the Department of Pathology, Liaquat University of Medical and Health Sciences, Jamshoro and Isra University Hospital, Hyderabad from May, 2014 to September, 2014. The study was approved by the ethical committee of Isra University, Hyderabad. A total of 145 newly diagnosed cases of CML were recruited in the study after obtaining the informed consent. All patients suffering from acute leukemia, polycythemia, essential thrombocythemia, myelofibrosis and multiple myeloma were excluded from the study. Bone marrow and blood samples were collected in sodium heparinized vacutainer using recommended techniques.

### Complete blood counting (CBC)

Complete blood counting (CBC) was performed using an automated hematoanalyzer (Sysmex XN 1000i).

### Cytogenetic analyses and Reverse transcription polymerase chain reaction (RT-PCR)

Cytogenetic analyses were performed by karyotyping according to the International System for Human Cytogenetic Nomenclature (ISCN) guidelines. All karyotype images were analyzed using the Cytovision software/workstation. This workstation is designed to allow time consuming metaphase capture to await import when convenient. This enables the use of sophisticated analysis and presentation tools available on the Cytovision. In order to identify BCR-ABL transcripts, reverse transcription polymerase chain reaction (RT-PCR) was performed using ipsogen BCR-ABL kit from QIAGEN. Details of the primers and PCR reactions are summarized in Table-I

**Table-IA:** Primer sequence for qualitative BCR-ABL determination;

Name of the primer	Sequence
BCR-e1	5’-ACCGCATGTTCCGGGACAAAAG-3’
BCR-b2	5’-ACAGAATTCCGCTGACCATCAATAAG-3’
BCR-rev	5’-ATAGGATCCTTTGCAACCGGGYCYGAA-3’
ABL-a2	5’-TGTTGACTGGCGTGATGTAGTTGCTTGG-3’

**Table-IB:** Composition of RT-PCR mix.

Component	Volume per sample (µl)	Final Concentration
RT Buffer, ×5	5.0	x1
dNTPs (10m M each)	2.0	0.8mM
Random nonamer (100µM)	5.25	21Um
RNAs Inhibitor (40U/µL)	0.5	0.8U/µl
Reverse Transcriptase (200U/µL)	1.0	8U/µl
DTT (supplied with Reverse Transcrpitase)	1.25	-
Heated RNA sample/control/IS-MMR Calibrator	10.0	40ng/µl
Final Volume	25.0	-

**Table-IC:** Temperature cycles for the PCR.

Reaction	Temperature
Reverse transcription 1	25 °C for 10 minutes
Reverse transcription 2	50 °C for 60 minutes
Inactivation	85 °C for 5 minutes
Cooling	4 °C for 5 minutes

### Statistical Analysis

The data were analyzed using SPSS version 21.0 (IBM, Corporation) and Microsoft excel. The continuous variables were presented as mean±SD and analyzed using student’s t-test. Categorical variables were analyzed by Chi-square test and results were presented as frequencies and percentages.

## RESULTS

The average age of recruited patients was 36±11.7 years. Of the 145 cases enrolled, most frequent age groups were 20-29.9 and 30-39.9 years (47.5% and 44.8%, respectively). Of the 145 investigated cases, a total of 109 (75.1%) were males and 36 (24.8%) were females (p=0.001). Anemia was noted in 74.4% and hematocrit of <20% in 46.2% of cases. Leukocytosis of >50,000/µL was noted in 68.5% of the total cases (Table-II).

**Table-II:** Clinico-pathological parameters of the study population (n=145).

Parameter	n (%)
WBC (/µL)	
4,000-11,000	46 (31.7)
<20,000	17 (11.7)
>150,000	82 (56.5)
RBC (millions/µL)	
<3.5	65 (44.8)
3.5-4.5	72 (49.6)
> 4.5	8 (5.5)
Hemoglobin (g/dl)	
< 5 g/dl	47 (32.4)
5-10 g/dl	61 (42.1)
10-13 g/dl	24 (16.5)
Platelet counts	
150-450,000	112 (77.2)
> 450,000	20 (13.8)
<50,000	13 (8.9)

**Fig.1:**
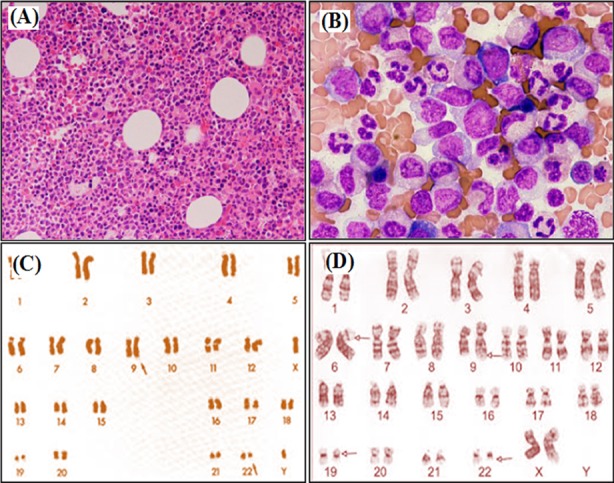
(A–B): Chronic myeloid leukemia (CML) showing hypercellular bone marrow on H & E staining at ×100 and ×400 magnification. (C): Classical karyotype of CML translocation – t(9; 22) & (D): CML Complex translocation 9,6,19,22.

Of the 145 patients, Ph+ chromosome was noted in 133 (91.7%) CML patients. Of the 133 Ph+ chromosome, standard chromosome was noted in 121 (90.9%), simple variant in 9 (6.7%) and complex variants were noted in 3 (2.2%) of cases (Table-III and Fig.1). All Ph+ patients showed BCR-ABL positivity. Of the 12 Ph- samples, 7 were BCR-ABL positive and 5 were BCR-ABL negative.

**Table-III:** Simple and complex variants of Philadelphia chromosome (n=12).

S. No	Variant Ph chromosome	Chromosome translocation
1	Simple variant Ph	46 xy t(16;22)
2	Simple variant Ph	46 xx t(19;22)
3	Simple variant Ph	46 xx t(13;22)
4	Simple variant Ph	46 xy t(17;22)
5	Simple variant Ph	46 xx t(11;22)
6	Simple variant Ph	46 xx t(18;22)
7	Simple variant Ph	46 xy t(15;22)
8	Simple variant Ph	46 xx t(14;22)
9	Simple variant Ph	46 x t(12;22)
10	Complex variant Ph	46 y t(6;9;22)
11	Complex variant Ph	46 x t(5;9;22)
12	Complex variant Ph	46 xx t(7;9;22)

**Fig.2:**
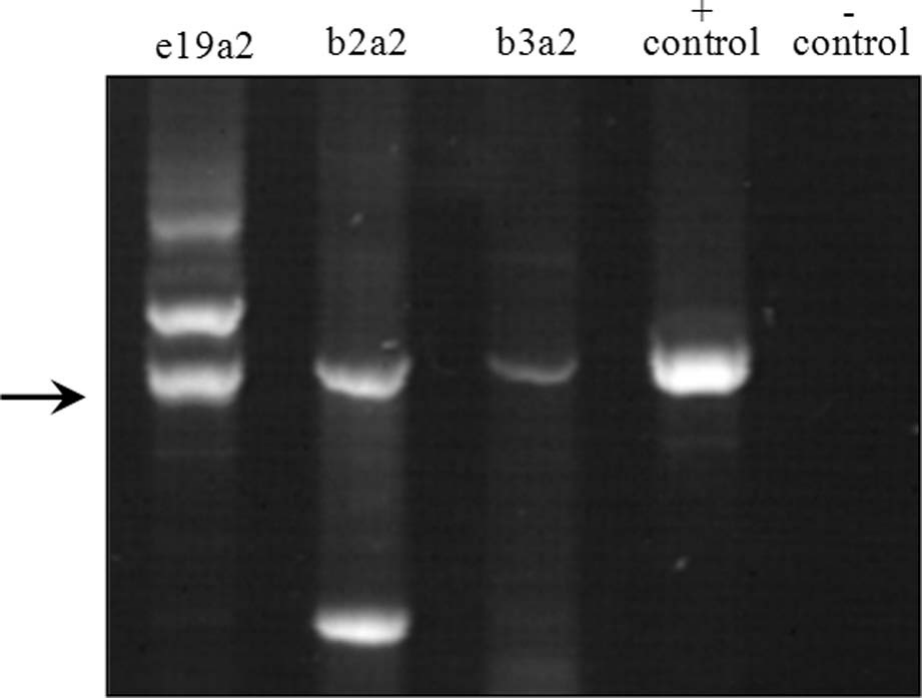
PCR gel electrophoresis to demonstrate bands indicating e19a2, b2a2 and b3a2 transcripts. Positive and negative controls are added in separate lanes.

Cytogenetic and molecular analyses of CML transcripts was conducted using RT-PCR (Fig.2). Our data show that the frequency of cytogenetic abnormalities b3a2, b2a2, b3a2+b2a2 and b3a2+e19a2 was 65, 24, 3 and 2 patients, respectively (Table-IV).

**Table-IV:** Cytogenetic and molecular analysis of transcripts in cases.

Transcript	Chronic Phase (n=130)	Accelerated Phase (n=9)	Blast Crisis (n=6)
b3a2	65	3	-
b2a2	24	-	1
b3a2+ b2a2	3	-	-
b3a2+ e19a2	2	-	-
b2a2+ e19a2	-	-	2

## DISCUSSION

RT-PCR is the most sensitive technique to detect various Ph chromosome transcripts expressed in CML patients. Moreover, there is panoply of data available to demonstrate the fact that expression of various Ph chromosome transcripts indicate different disease outcome. Moreover, RT-PCR is an extremely sensitive tool to monitor disease progression even in the absence of detectable morphology using bone marrow examination. However, local data for Ph chromosome variants in Pakistani CML patients are scanty.

In the study described herein, Ph chromosome was detected in 91.7% of CML patients. These findings are consistent with previous studies which have reported Ph chromosome in 90-95% of cases.^13^ Additional cytogenetic abnormalities were detected in 70% of cases in the present study as shown in Table-IV.

In our study, most frequent cytogenetic abnormalities were b3a2, b2a2 and b3a2+b2a2 found in 70%, 34% and 2% of cases respectively (Table-IV). Several local and international studies have reported results similar to our findings with b3a2 being the most common transcript amplified in CML patients. Iqbal 14 for example reported that of the 130 Pakistani CML patients investigated, a total of 63.8% expressed b3a2.

Similarly, Ruiz-Argüelles *et al.*^15^ investigated a total of 238 Mexican Mestizos patients with Ph positive CML. Of these, 54.2% showed b3a2 subtype, 43.2% b2a2 and 2.5% b3a2/b2a2. Similarly, De Lemos *et al*.^16^ from Brazil performed RT-PCR for *BCR-ABL* in 22 CML patients. Of these patients, 15 (68%) were in chronic phase, five (23%) in accelerated phase and two (9%) in blastic phase; 59% patients had b3a2 and 41% had b2a2 transcripts. However, Paz-y-Miño *et al*.^17^ found that the b3a2 transcript is 5.4% frequent while a total of 94.6% were b2a2 transcripts in Ecuadorian Mestizos CML patients. These findings are however, in contrast to our study (Table-III). There could be several reasons to explain these contradictory findings including our unique genetic make-up of CML.

In summary, the present study reports Ph, its variants and cytogenetic abnormalities in Pakistani CML patients. Strength of present study lies in its prospective design, inclusion and exclusion criteria and its contribution towards establishment of local data. Limitations of present study may be small sample size and we could not analyze all of cytogenetic abnormalities due to lack of modern laboratory facilities and funding issues. Moreover, further studies correlating expression patterns of various Ph chromosome transcripts with clinical parameters (such as disease progression and patients’ outcome) would further delineate the role of detecting such transcripts in CML patients.

## CONCLUSION

The study presented herein reports frequency of the Ph to be of 90.9% in CML patients using a highly sensitive technique, the RT-PCR. Cytogenetic abnormalities are reported to be at a lower frequency, similar to the studies from other countries. Cytogenetic and molecular studies must be conducted for better management of CML cases in our locality. Overall, the study has contributed towards the current understanding of cytogenetic and molecular abnormalities in CML cases, particularly in establishment of local data pool.
